# Different Effects of *Metarhizium anisopliae* Strains IMI330189 and IBC200614 on Enzymes Activities and Hemocytes of *Locusta migratoria* L.

**DOI:** 10.1371/journal.pone.0155257

**Published:** 2016-05-26

**Authors:** Guangchun Cao, Miao Jia, Xia Zhao, Lei Wang, Xiongbing Tu, Guangjun Wang, Xiangqun Nong, Zehua Zhang

**Affiliations:** State Key Laboratory for Biology of Plant Diseases and Insect Pests/Institute of Plant Protection, Chinese Academy of Agricultural Sciences, Beijing, P. R. China; Institute of Plant Physiology and Ecology, CHINA

## Abstract

**Background:**

Metarhizium is an important class of entomopathogenic fungi in the biocontrol of insects, but its virulence is affected by insect immunity. To clarify the mechanism in virulence of Metarhizium, we compared the immunological differences in *Locusta migratoria* L. when exposed to two strains of *Metarhizium anisopliae* (Ma).

**Results:**

The virulence of Ma IMI330189 was significantly higher than that of Ma IBC200614 to locust, and IMI330189 overcame the hemocytes and began destroying the hemocytes of locust at 72 h after spray, while locust is immune to IBC200614. IMI330189 could overcome the humoral immunity of locust by inhibiting the activities of phenol oxidase (PO), esterases, multi-function oxidases (MFOs) and acetylcholinesterases in locust while increasing the activities of glutathione-S-transferases (GSTs), catalase and aryl-acylamidase (AA). However IBC200614 inhibit the activities of GSTs and AA in locust and increase the activities of MFOs, PO, superoxide dismutase, peroxidase and chitinase in locust. The changes of enzymes activities in period of infection showed that the time period between the 2^nd^ and the 5^th^ day after spray is critical in the pathogenic process.

**Conclusion:**

These results found the phenomenon that Ma initiatively broke host hemocytes, revealed the correlation between the virulence of Ma and the changes of enzymes activities in host induced by Ma, and clarified the critical period in the infection of Ma. So, these results should provide guidance for the construction of efficient biocontrol Ma strains.

## Introduction

The growing demand to reduce chemical inputs in agriculture, along with the increased resistance to insecticides, has provided great impetus for the development of alternative forms of insect-pest control. Metarhizium offers an attractive alternative to the use of chemical pesticides [1]. However, in the process of evolution, insects have developed a defensive system to effectively avoid infection by entomopathogenic fungi [2–6]. The thickness, structure, and chemical composition of the cuticle are important in preventing the adhesion and germination of conidia, and the penetration of fungal hyphae [7, 8]. The rapid healing of wounds to the cuticle is also important in preventing the penetration of fungi [9]. It has been documented that the differences in the cuticle of locusts and cockroaches led to differences in the germination and penetration abilities of *Metarhizium anisopliae* and *Metarhizium acridum*; therefore, the two fungi had significantly different virulence levels in locusts and cockroaches [10]. In hemolymph, the hemocytes and plasma played important roles in eliminating the fungi that are present in the hemolymph, and in eliminating the destructive toxins that are secreted by fungi [8, 11]. Many enzymes are involved in these mechanisms and play important roles in the defense of insects against fungal infections [11–17]. However, a difference in the virulence of a fungus that was induced by adaptation to the host’s hemolymph was not found.

During evolution, fungi have developed mechanisms to overcome the immune systems of insects. The first mechanism involves evading the host immune system by changing their cell surface morphology and formation. For example, entomopathogenic fungi have cells with different morphologies, including no cell wall or almost no cell wall in the host hemolymph. These forms aid in the dispersion and clustering of entomopathogenic fungi in the hemocoel and can increase the surface area to absorb more nutrients. Conversely, the lower level of β-1,3-glucan in the cell surface does not stimulate the host immune response [10, 18]. The second mechanism disables the host immune response by secreting extracellular toxins, such as destruxins [19–21], cyclosporine [22], beauverolide [23] and peptide alkaloids efrapeptin [24]. Thus, the adaption to the host’s hemolymph is important for the virulence of the fungus to the insect. However, the different mechanisms necessary to overcome the host’s immune system have not been studied by comparing the disease efficacy of different strains of Metarhizium.

Multi-function oxidases (MFOs), esterases (ESTs) and glutathione-S-transferases (GSTs) are three important families of enzymes in insects that participate in the detoxification of various xenobiotics and insecticides, and play important roles in the resistance of insects to various insecticides [25]. MFOs can mediate resistance to all classes of insecticides by degradation because of their genetic diversity, broad substrate specificity and catalytic versatility [26]. GSTs play a pivotal role in cellular antioxidant defenses against oxidative stress by conjugating reduced glutathione to the electrophilic centers of natural and synthetic exogenous xenobiotics and can mediate resistance to organophosphate (OP), organochlorines and pyrethroids [27–29]. ESTs perform important functions in the insect by catabolizing the esters of higher fatty acids, which proceeds activity in the flight muscles and enables insects to fly, by mobilizing lipids, including those of the fat body [30], by degrading inert metabolic esters, including various xenobiotics [31], and by forming resistance to OP, carbamates and pyrethroids [25]. The degradation of toxic molecules with ESTs and GSTs during infection has a key role in protecting insects from pathogens [32], *Beauveria bassiana* spores and its secondary metabolite increase ESTs and GSTs activities in the hemolymph of infected and treated adults of *Eurygaster integriceps*[33]. However, with the development and application of new insecticides, arylamidase (AA) was found to act against anthranilic diamide-based insecticides and non-steroidal ecdysone agonists [34, 35], and chitinase (CHI) was found to act against non-steroidal ecdysone agonists [34]. Thus, clarification of the roles of these enzymes in insect defense responses is important for the efficient use of Metarhizium.

The main role of PO in melanogenesis is to convert phenols to quinones that subsequently polymerize to form melanin [36]. Thus, increased PO activity can strengthen the immune system’s capability to challenge xenobiotics and healing in insects. The activity of PO might be altered by trichlorfon in *Macrobrachium rosenbergii* and might be increased by butane-fipronil resistance in the diamondback moth [37, 38]. Phenol oxidase (PO) is an important tool against several pathogens [39]. After topical application of conidia from Met 189, no activation of prophenoloxidase is detected, but injection of blastospores from Met 189 brings about a transient increase in phenoloxidase activity in the haemolymph in both adult locusts and 5th instar nymphs [40]. In desert locust, the activity of the enzyme, phenoloxidase, decreased during the course of infection by *M*. *anisopliae*[41]. Analysis of PO activities revealed that by 48–60 hr post challenge the PO titers in the HL (hemocyte lysate) sampled from infected larvae decreased seven-fold, whereas plasma PO titers increased [42]. However, the positive relationship between PO activity and successful pathogen defense has not found broad support because many studies failed to estimate the severity of the pathogen challenge used, such as failing to measure the median lethal dose, or did not consider that PO may be used for other functions besides immunity, such as egg production, pigment synthesis, molting or sclerotization. Additionally, many studies did not consider that PO may only be activated for short periods of time in specific tissues and also acts in different ways depending on the immune challenge [43], and many studies did not consider that the use of dead pathogens may trigger different responses than live pathogens [44,45]. Clarifying the role of PO in the immune response of insects to fungi is important in the efficient use of entomopathogenic fungi as biocontrol agents.

Insects possess a suite of antioxidant enzymes, including catalase (CAT), superoxide dismutase (SOD) and peroxidase (POD), which may form a concatenated response to the onslaught of dietary and endogenously produced oxidants [46, 47]. SOD catalyzes the reaction of O_2_ to H_2_O_2_, and then H_2_O_2_ is removed by POD, which needs a substrate that becomes oxidized, and CAT, which does not need a substrate that becomes oxidized [48]. The activities of these enzymes can be induced by insecticides, and the increases in the activities of these enzymes were related to the resistance of insects to pesticides [49, 50]. In American white shrimp, SOD is a modulator of immune function, and its activity was significantly related to the melanization and phagocytotic abilities of insects [51]. In *Periplaneta Americana*, *M*. *anisopliae*, *Isaria fumosoroseus* and *Hirsutella thompsonaii* fungal infections initiate oxidative stress in the midgut, fat body, whole body and hemolymph, and decrease the CAT activity of fat body and midgut [52]. In *Spodoptera litura*, the activities of SOD, CAT, POD and specific ascorbate peroxidase in the larval body decreased after it was treated with crude destruxins at the doses that caused 90% mortality[53].Thus, the clarification of the roles of these enzymes in the immune response of insects to fungi is important for the efficient use of entomopathogenic fungi as biocontrol agents.

In this paper, the virulence of two fungal strains of *M*. *anisopliae* to *Locusta migratoria* L. was compared using a *L*. *migratoria* strain that had been reared for 11 generations from one oocyst in the laboratory. As observed by blood smears, the two strains of *M*. *anisopliae*, IMI330189 and IBC200614, displayed different abilities to adapt to the immune responses in the hemocytes of *L*. *migratoria*. The activities of the above enzymes in *L*. *migratoria* were compared after it was treated with IMI330189 and IBC200614 to speculate which enzymes were involved in the immune response of *L*. *migratoria* to *M*. *anisopliae*. These results can provide direction for future research to enhance the construction of toxic strains of Metarhizium.

## Materials and Methods

### Insects

*L*. *migratoria* were obtained from a laboratory colony that was established in 2007 from eggs collected from Hebei Province, China, and had been reared for 11 generations from one oocyst in the laboratory, without exposure to insecticides. The location (N38°30'33.46", E117°25'32.85") which is covered with saline-alkali soil is nearby the Bohai Sea. We have got the permission for us to conduct the field studies by Cangzhou Academy of Agriculture and Forestry Sciences of Hebei province, who is the authority department responsible for pest control in agriculture and forestry land, also with the protection of wildlife in Cangzhou. With the help of Dr. Qinglei Wang (Cangzhou Academy of Agriculture and Forestry Sciences), we collected eggs in Autumn for our laboratory experiment. This location is a natural ecosystem, it is not involving endangered or protected species during the field studies. The colony was cultured on wheat seedlings and an artificial diet in the laboratory. The nymphs were reared in a growth chamber, and then transferred to cages (60 cm long × 50 cm wide × 70 cm high) in a chamber maintained at 60 ± 5% RH, 30 ± 2°C and 14:10 (L:D).

### Fungal cultures

*M*. *anisopliae* strains IMI330189 was generous gifts from CABI biosciences, and IBC200614 was the serial number in our laboratory to save ATCC200614 that was purchased from ATCC (American Type Culture Collection). Two strains were selected based on their similar capabilities in penetrating the cuticle of *L*. *migratoria* when similarity of Mos1 gene for Metarhizium osmosensor-like protein of two strains was 92%, while they have different in morphology of their conidia and conidiophores and virulence to *L*. *migratoria* in previous experiment. Two funguses were cultured on potato dextrose agar (PDA) in Petri dishes at 28°C for 7–10 days under constant light, and were subcultured every 14 days. Conidia harvested from culture plates by scraping the surface of the PDA with a sterile mounted needle were collected into plastic centrifuge tubes containing 0.1% Tween 80 sterile water, and then an oscillator was used to break up any aggregates. The concentration of spores was determined using an improved Neubauer hemocytometer and adjusted to a concentration of 3.34 × 10^8^ spores mL^−1^. This procedure was repeated so that a fresh suspension of spores was used for each experiment.

### Reagents

The measure kits for measuring SOD, POD and CAT activities were purchased from Nanjing Jiancheng Bioengineering Institute (Nanjing, China). 5,5'-Dithiobis-(2-nit-robenzoic acid) (DTNB), acetylthiocholine iodide (ATChI), Catechol, Coomassie brilliant blue G-250, 1-chloro-2,4-dinitrobenzene (CDNB) and 1,2-dichloro-4-nitrobenzene (DCNB), 1-Naphthyl acetate (α-NA), 4-nitroanisole (p-NA) and NADPH were obtained from Sigma (St. Louis, MO, USA); glutathione (GSH), Fast Blue RR salt and Bovine serum albumin were purchased from Pierce (Rockford, IL, USA).

### Bioassay

The conidia of IMI330189 and IBC200614 were dried and preserved. The solution concentrations of the two strains were predicted by spore content and germination rate.

Five conidial suspensions in sterile water containing 0.1% Tween 80 (as wetting agent) [54] were prepared at concentrations from 3.34 × 10^3^ to 3.34 × 10^7^ spores mL^−1^. Sterile water containing 0.1% Tween 80 was used as the negative control. Third instar nymphs were identified by size and selected. A total of 45 third instar nymphs were treated with each spore concentration with potter spray tower, and three replications were performed. All of the nymphs were fed wheat seedlings. Mortality was recorded at 24-h intervals for 7 d.

### Collection and treatment of hemolymph

Hemolymph was taken from the insects at 24-h intervals during infection and blood smears were made. Hemolymph was collected from the arthrodial membrane of the hindleg of the locust (control group and treated group). The membrane was first swabbed with 70% ethanol, allowed to air dry and then pierced with a sterile needle. The hemolymph was collected using a 10 μL Eppendorf Pipetman over ice to prevent coagulation. The hemolymph was dripped on slide glass, and then smeared. The smears air dried 10 min, and a drop of Giemsa dye (0.5 g Giemsa and 22 mL glycerine, thoroughly mixed) was added. After 2 h incubation at 56°C, 33 mL pure methanol was added. A prepared liquid of Giemsa dye: Sorensen buffer (1:9; pH 6.81) was placed in a wet box and slides were stained for 30 min. The excess liquid was removed and slides were rinsed with tap water. They were then returned to the wet box, covered with Sorensen buffer (50 mL 1/15M Na_2_HPO_4_ + 50 mL 1/15M KH_2_PO_4_, pH 6.81) and left for 25 min. The excess liquid was removed, and slides were washed with sterile water and xylene, and then left to air dry. They were then mounted with neutral balata. The blood nucleus was dyed red or blue, while the cytoplasm was more transparent. Slides were observed and photographed under the microscope (Olympus BX61 DP72).

### Enzyme preparation

The whole bodies of third instar nymphs for each treatment were homogenized in 0.1 mol L^−1^ Na-phosphate buffer (PB) at pH 7 (for EST, 1.5 mL PB with 0.1% TritonX-100; for AChE and PO, 1.5 mL PB), pH 7.3 (for MFO, 1 mL PB with 1 mmol L^-1^ EDTA and 1 mmol L^−1^ DTT; for SOD, POD and CAT, 1.5 mL PB) and pH 7.5 (for GST, 1.5 mL PB). Extracted samples were centrifuged at 10,000 ×g for 10 min at 4°C. Supernatants were transferred to new Eppendorf tubes and centrifuged at 15,000 ×g for 20 min at 4°C. Then, the supernatants were used to determine the enzymes activities and protein concentrations. 10 nymphs were tested for each enzyme.

### Enzyme activity assays

#### ESTs

ESTs activity was assayed using the method of Han [55], with modifications. In brief, 100 μL of 1-naphthyl acetate solution (10 mM), 100 μL of fast blue RR Salt solution (1 mM), and 90 μL of PBS was added into each microplate well. The enzymatic reaction was initiated by the addition of 10 μL of the enzyme preparation. The reaction was measured at 450 nm every 25 s for 10 min at 37°C. Softmax Pro 6.1 (Molecular Devices, USA) was used to record and analyze the readings. The enzyme activity was measured as the absorbance change rate per min (mOD min^−1^)

#### GSTs

The method of Oppenoorth was used with modifications [56]. When the substrate was CDNB, 10 μL aliquots of samples were added to microplate wells containing 90 μL PB, then 100 μL 1.2 mmol L^−1^ CDNB and 100 μL 6 mmol L^−1^ GSH were added. When DCNB was the substrate, 50 μL aliquots of samples were added to microplate wells, and then 100 μL 1.2 mmol L^−1^ DCNB and 100 μL 6 mmol L^−1^ GSH were added. The reactions were measured at 340 nm every 25 s for 10 min at 27°C. Softmax Pro 6.1 was used to record and analyze the readings. For both enzymes, the enzyme activities were measured as the absorbance change rate per min (mOD min^-1^)

#### MFOs

MFOs activity was assayed using the method of Hansen and Hodgson with modifications [57]. In each microplate well, 100 μL 2 mM p-NA solution and 50 μL enzyme stock solution were mixed. The reaction was initiated by addition of 10 μL of 9.6 mM NADPH. The reaction was measured at 405 nm every 25s for 10 min at 27°C. Softmax Pro 6.1 was used to record and analyze the readings. The enzyme activity was measured as the absorbance change rate per min (mOD min^−1^).

#### Acetylcholinesterase (AChE)

AChE activity was assayed according to the method of Han [55], with modifications. Each microplate well contained a mixture of 50 μL enzyme stock solution, 50 μL PB, 100 μL 45 μmol L^−1^ DTNB and 100 μL 1.5 mmol L^−1^ ATChI. The reaction was measured at 405 nm every 30 s for 40 min at 27°C. Softmax Pro 6.1 was used to record and analyze the readings. The enzyme activity was measured as the absorbance change rate per min (mOD min^−1^)

#### PO

PO activity was assayed using the method of Luo [58], with modifications. Aliquots (20 μL) of samples were added to microplate wells containing 180 μL 10 mmol L^−1^ catechol. The reaction was measured at 420 nm every 1 min for an hour at 27°C. Softmax Pro 6.1 was used to record and analyze the readings. The enzyme activity was measured as the absorbance change rate per min (mOD min^−1^)

#### CAT, SOD and POD

The activities of CAT, SOD and POD were determined using the appropriate assay kits (Nanjing Jiancheng Bioengineering Institute, Nanjing, China), according to the manufacturer’s instructions. The enzyme activities were measured as μmol/min/mg, μmol/min/mg and μmol/min/g, respectively.

#### CHI

CHI activity was measured by a reducing-sugar assay. The reaction mixture (400 μL) containing 300 μL 1% (w/v) colloidal chitin and 100 μL aliquots of samples [59], was incubated at 37°C in Eppendorf tubes. After 4 h of incubation, the mixture was centrifuged at 8,000 ×g for 5 min to precipitate the remaining chitin. Supernatant (200 μL) was mixed with 80 μL 0.8 mol L^−1^ (pH 9.1) potassium tetraborate, and the reaction was terminated by boiling at 100°C for 5 min. It was then cooled to room temperature by running water. The mixture was mixed with 1.2 mL DMAB (10 g DMAB diluted into 1 L distilled water, and was then diluted 10-fold with glacial acetic acid when it was used) incubated at 37°C for 20 min, and then was cooled to room temperature by running water. The absorbance was detected at 585 nm. The enzyme activity was measured as the absorbance change rate per mg protein (mmol/min/mg).

#### Aryl-acylamidase (AA)

AA activity was measured as described previously with minor modifications [34]. In each assay, one larva was homogenized in 1.5 mL ice-cold phosphate buffer (0.05 M, pH 7.5). The homogenate was centrifuged at 15,000 ×g at 4°C for 20 min. The resulting supernatant was used as the enzyme source. The reaction mixture, including 50 μL enzyme source, 50 μL 1.2 mM p-nitroacetanilide (dissolved in absolute ethanol) and 150 μL phosphate buffer (0.05 M, pH 7.5) was incubated in a water bath at 35°C for 30 min. The reaction was stopped by boiling in water for 10 min, and the mixture was then centrifuged at 10,000 ×g for 15 min. The released p-nitroaniline in the supernatant was measured at 405 nm, and the boiled enzyme was used as a control. The enzyme activity was measured as the absorbance change rate per mg protein (mmol/min/mg).

#### Protein assay

The protein concentrations of the samples were determined by Bradford’s method [60] and measured at 595 nm. Bovine serum albumin was used to build a calibration curve. Softmax Pro 6.1 was used to record and analyze the readings.

### Data Analysis

For bioassays, the median lethal concentration (LC_50_) and the slope of the concentration-mortality curve for each assay were estimated by probit analysis [61] using POLO-PC software [62]. One-way ANOVAs were also used to analyze protective enzymes (SOD, CAT and POD), detoxification enzymes (EST, GST and MFO) and PO, AChE, CHI and AA activities in the nymphs of *L*. *migratoria* that were treated with different concentrations of *M*. *anisopliae*. Differences among means were compared using the least significant difference test at *P* < 0.05.

## Results

### The virulence of IMI330189 and IBC200614 to *L*. *migratoria*

The virulence of IMI330189 and IBC200614 to *L*. *migratoria* was bioassayed, and the results are shown in Table 1. The bioassay results showed that the virulence of *M*. *anisopliae* strains IMI330189 and IBC200614 to *L*. *migratoria* were significantly different. At the 3.34 × 10^7^ concentration, IMI330189 killed about 50% of *L*. *migratoria* by the 7^th^ day, with a deduced LC_50_ of 1.98 × 10^7^ at the 7^th^ day after sprayed. However, IBC200614 only killed about 20% of *L*. *migratoria manilensis* by the 7^th^ day. IMI330189 killed *L*. *migratoria* in a concentration-dependent and time-dependent manner, but this was not obvious in IBC200614. The muscardine cadaver rates were 95.83% and 57.24% in the populations that were killed by IMI330189 and IBC200614, respectively.

**Table 1:** Virulence of *M*. *anisopliae* strains IMI330189 and IBC200614 to *L*. *migratoria*.

	Concentration of *M*. *anisopliae*	Treatment time (d)			
		4	5	6	7
IMI330189	3.34 x10^3^	10.00±0.00	13.33±0.03	23.33±0.03	23.33±0.03
	3.34 x10^4^	20.00±0.06	23.33±0.09	30.00±0.06	36.67±0.07
	3.34 x10^5^	16.67±0.03	30.00±0.06	30.00±0.06	36.67±0.03
	3.34 x10^6^	6.67±0.07	20.00±0.10	36.67±0.03	43.33±0.03
	3.34 x10^7^	10.00±0.10	20.00±0.10	30.00±0.00	50.00±0.06
CK		3.33±0.03	3.33±0.03	3.33±0.03	3.33±0.03
IBC200614	3.34 x10^3^	3.33±0.03	6.67±0.03	6.67±0.03	6.67±0.03
	3.34 x10^4^	10.00±0.00	13.33±0.03	13.33±0.03	13.33±0.03
	3.34 x10^5^	6.67±0.03	6.67±0.03	10.00±0.00	10.00±0.00
	3.34 x10^6^	3.33±0.03	3.33±0.03	6.67±0.03	13.33±0.03
	3.34 x10^7^	3.33±0.03	6.67±0.03	16.67±0.03	16.67±0.03

### IMI330189 and IBC200614 colonization in the hemolymph of *L*. *migratoria*

**Fig 1:**
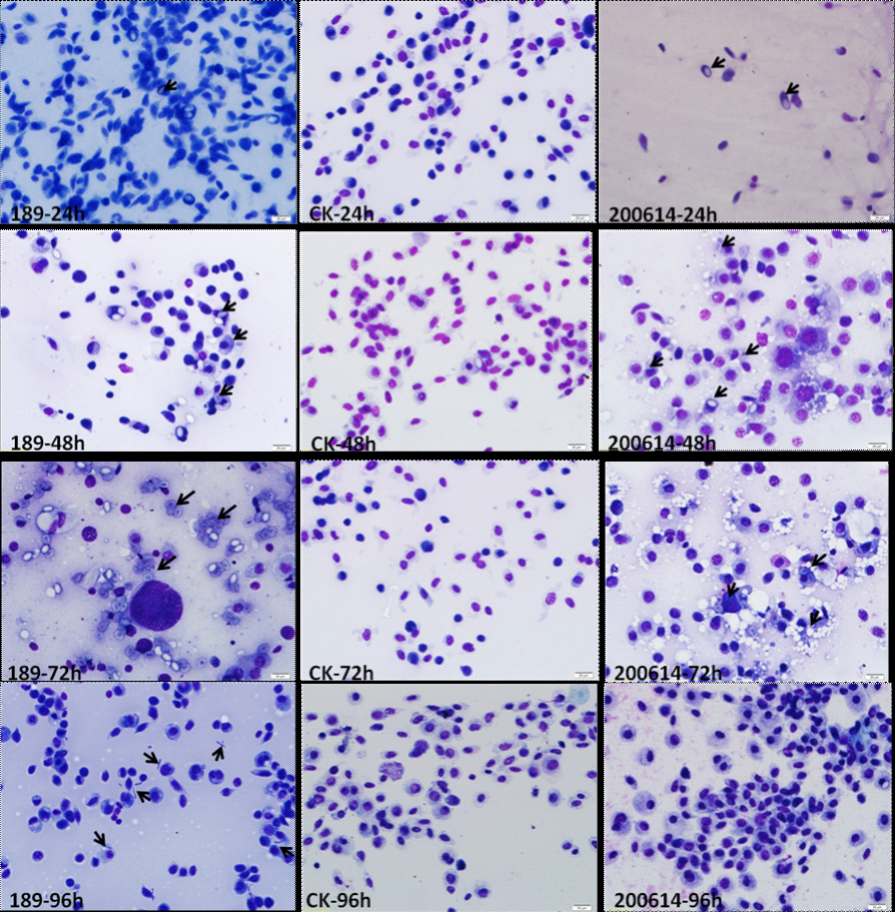
Growth of *M*. *anisopliae* strains IMI330189 (189) and IBC200614 (200614) in the hemolymph of *L*. *migratoria* during different periods. Note: In the images at 24, 48 and 72 h after infection, the arrows point to the yeast-like cells. In the image taken at 96 h, the arrows point to the fungal hyphal bodies. Scale bar = 20 μm.

**Fig 2:**
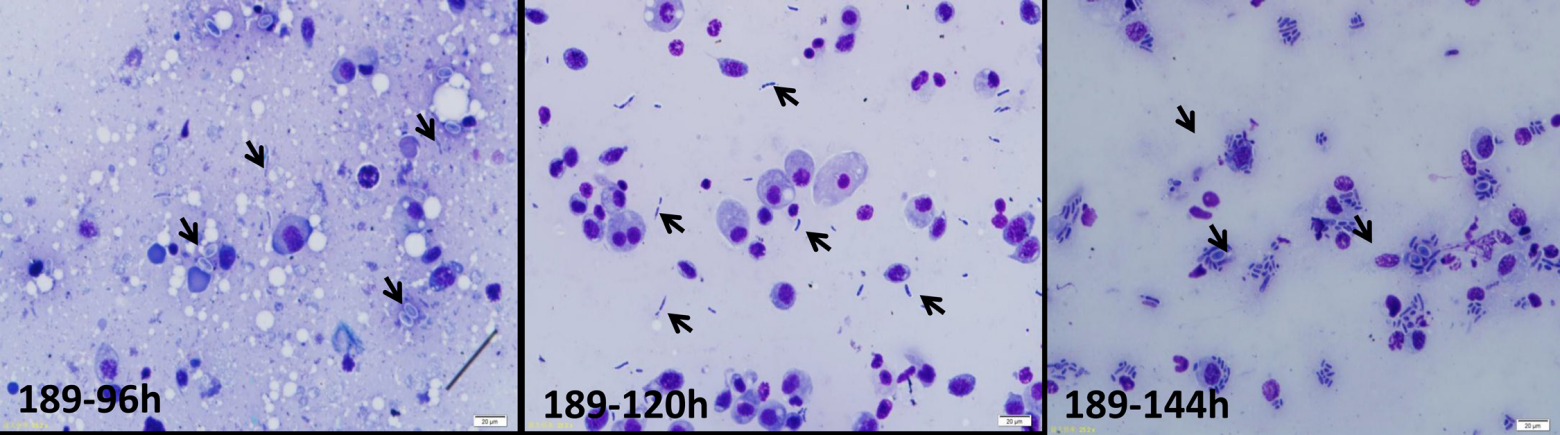
Mycelial segment treated by *M*. *anisopliae* strain IMI330189 (189). Note: The arrows point to the fungal hyphal bodies, bar = 20 μm.

To observe the IMI330189 and IBC200614 strains’ colonization in the hemolymph of *L*. *migratoria*, we produced a series of hemolymph smears. We found the yeast-like cells of *Metarhizium* and vacuoles in plasmatocyte in the hemolymph of *L*. *migratoria* 24–48 h after inoculation, (Fig 1). At 72 h after inoculation, the yeast-like cells had significantly increased, and at 96 h after inoculation, hyphal bodies (short fragments of hyphae) were only found in the hemolymph of *L*. *migratoria* that was infected by IMI330189. Thereafter, the hyphal bodies were significantly increased at 120 h and crowded the hemocoel of *L*. *migratoria* at 144 h (Fig 2). Thus, *L*. *migratoria* was killed by IMI330189. However, the hyphal bodies of IBC200614 were not found at 96 h, 120 h and 144 h after inoculation of IBC200614, and *L*. *migratoria* was not killed by IBC200614.

### Multiplication of IMI330189 in the hemolymph of *L*. *migratoria*

**Fig 3:**
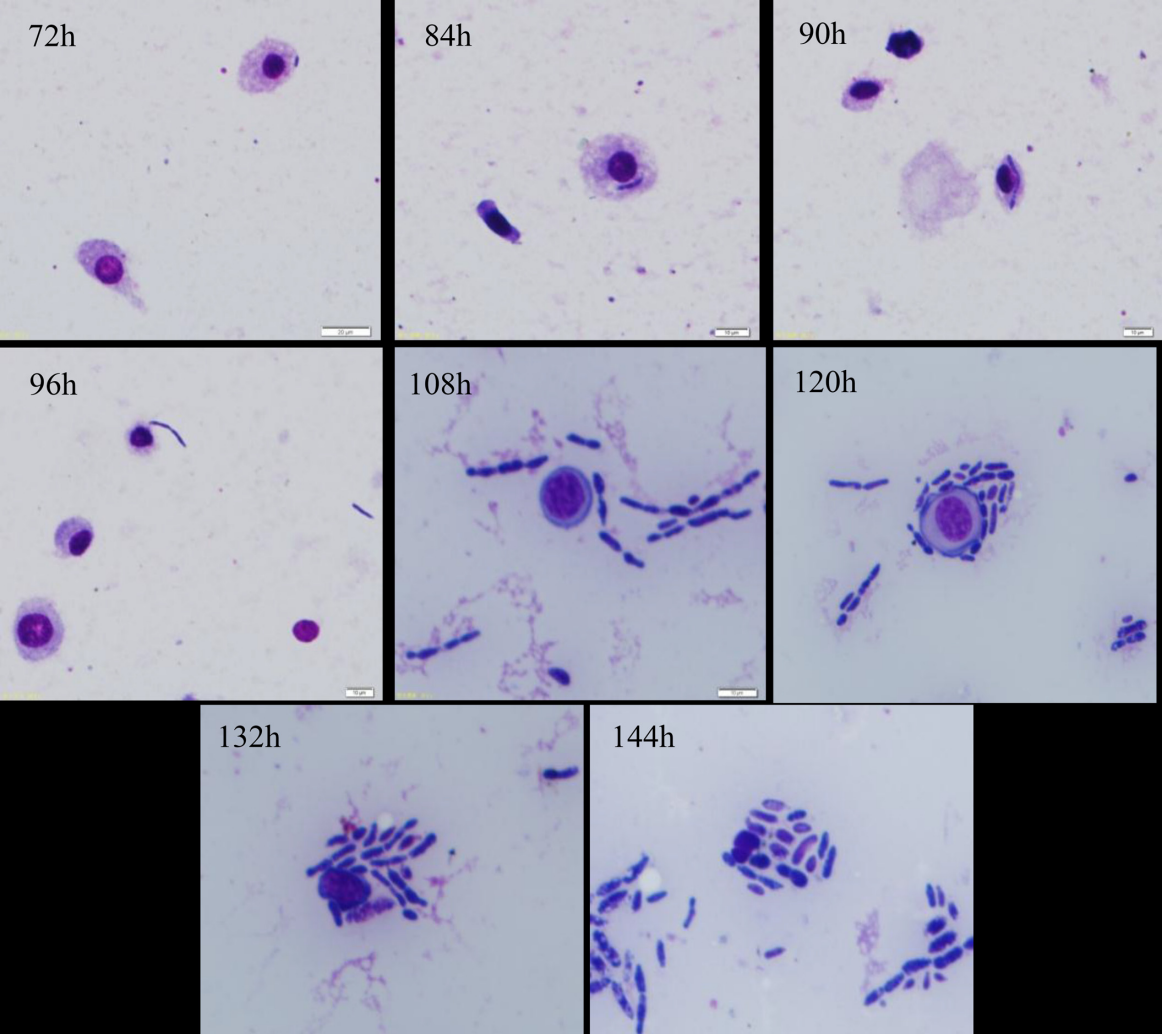
Mycelium segments that were phagocytized by granulocytes and mycelium growth.

To clarify the process of IMI330189 to destroy the hemocytes of *L*. *migratoria*, we shortened the interval of sampling times beginning at 72 h post-IMI330189 infection, and the results are shown in Fig 3. At 84 h, hyphal bodies of IMI330189 were swallowed by granulocytes of *L*. *migratoria*, and at 90 h, hyphal bodies of IMI330189 were sired in granulocytes of *L*. *migratoria*. At 96 h, hyphal bodies of IMI330189 broke through the granulocytes of *L*. *migratoria* and were released into the hemolymph. At 108 h, hyphal bodies of IMI330189 aggregated and damaged hemocytes of *L*. *migratoria* to proliferate itself.

### The concentration-dependent effect of IMI330189 and IBC200614 to the activities of enzymes in *L*. *migratoria*

**Fig 4:**
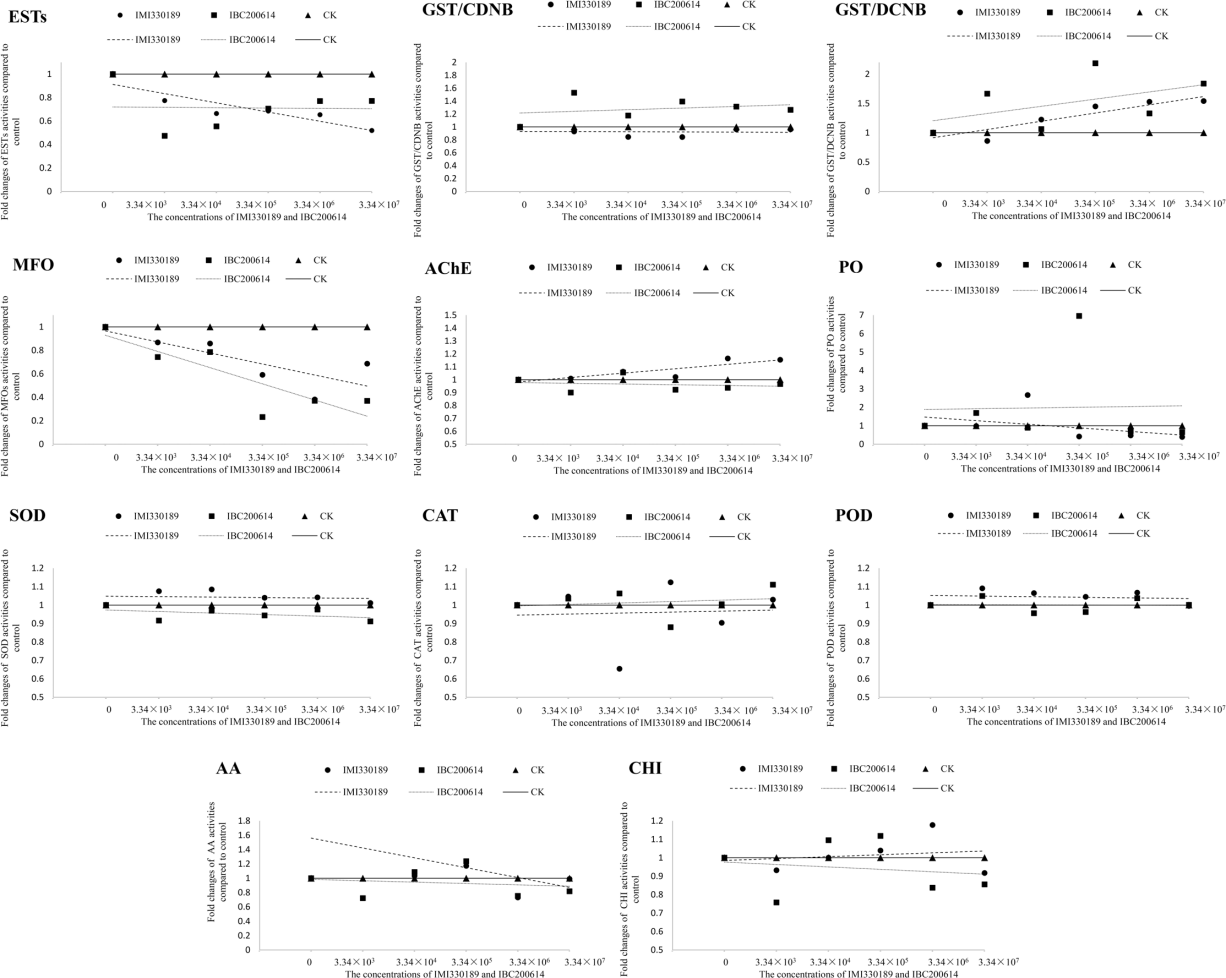
Fold changes in the activities of enzymes of *L*. *migratoria* on the 7^th^ day after it was treated by different concentrations of *M*. *anisopliae* strains IMI330189 and IBC200614.

The activities of enzymes in *L*. *migratoria*, which was infected by various concentrations of IMI330189 and IBC200614, were detected, and the results compared with controls were showed in Fig 4 and S1 Table. These results showed that the activities of ESTs, GSTs, MFOs, AChEs, PO, SOD, POD, CAT and AA in *L*. *migratoria* were significantly affected, while the activity of CHI was not significantly affected by IMI330189 and IBC200614. The trend line was used to analysis the difference in the effect of IMI330189 and IBC200614 to the activities of enzymes in *L*. *migratoria*, and the results were showed in Fig 4. The concentration-dependent in the effect of IMI330189 and IBC200614 to the activities of enzymes in *L*. *migratoria* was analyzed, and the results were showed in S2 and S3 Tables, respectively.

From the results in Fig 4, S2 and S3 Tables, we could find that the activities of ESTs were inhibited by IMI330189 and IBC200614 (*P*<0.05); the activities of GSTs/CDNB were slightly inhibited by IMI330189, while they were increased by IBC200614; the activities of GSTs/DCNB was increased by IMI330189 with a concentration-dependent manner (*P*<0.05), while they were increased more by IBC200614; the activities of MFOs were inhibited by IMI330189, while they were lightly inhibited by IBC200614; the activities of AChEs were increased by IMI330189, while they were lightly inhibited by IBC200614; the activity of PO was inhibited by higher concentration of IMI330189, while it was increased by IBC200614 and lower concentration of IMI330189; the activity of SOD was increased by IMI330189 with a concentration-dependent manner(*P*<0.05), while it was inhibited by IBC200614; the activity of CAT was inhibited by IMI330189, while it was increased by IBC200614; the activity of POD was hardly affected by both strains; the activity of AA was increased by the lower concentration of IMI330189, while it was inhibited by IBC200614 and the higher concentration of IMI330189.

### The time-dependent effect of IMI330189 and IBC200614 to the activities of enzymes in *L*. *migratoria* in the process of infection

**Fig 5:**
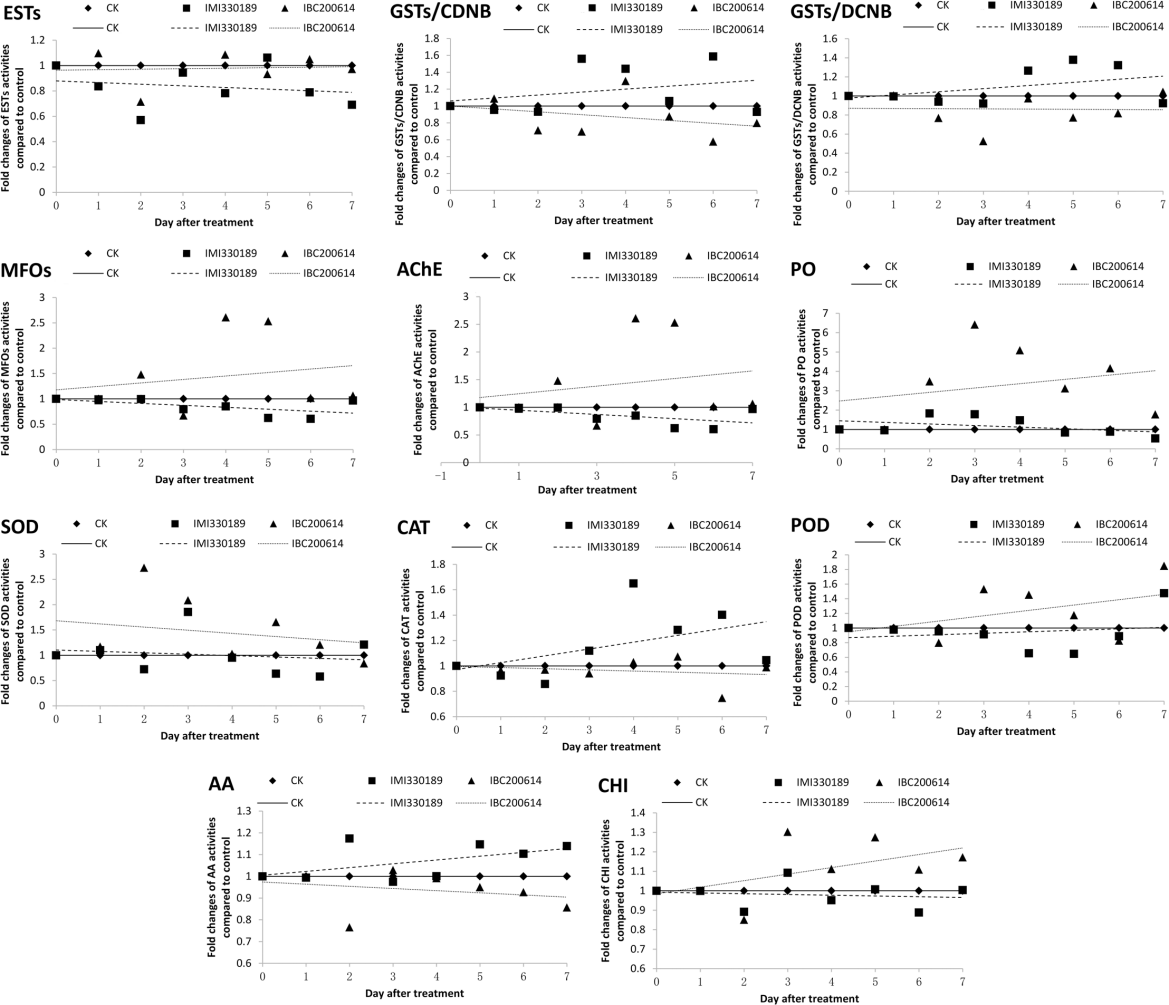
Fold changes in the activities of enzymes of *L*. *migratoria* during the infection process of *M*. *anisopliae* strains IMI330189 and IBC200614 at similar concentrations.

To clarify the functions of enzymes in the immune response of *L*. *migratoria* to *M*. *anisopliae*, we measured the activities of enzymes in *L*. *migratoria* during infection by IMI330189 and IBC200614, and the results are shown in Fig 5 and S4 Table. The trend line was added into Fig 5 to help us analyze the difference in the effect of IMI330189 and IBC200614 to the activities of the examined enzymes in *L*. *migratoria* in the process of infection.

From Fig 5, we found that ESTs activities were inhibited by IMI330189 with the advance of infection, while they were not significantly affected by IBC200614; the activities of GSTs/CDNB and GSTs/DCNB were increased by IMI330189 with the advance of infection, while they were inhibited by IBC200614; the activities of MFOs and AChEs were inhibited by IMI330189 with the advance of infection, while they were increased by IBC200614; the PO, SOD, POD and CHI activities were not significantly affected by IMI330189, while they were significantly increased by IBC200614 with the advance of infection; the CAT and AA activities were significantly increased by IMI330189 with the advance of infection, while it was not significantly affected by IBC200614.

## Discussion

Insect possess a systematic and comprehensive defense system, and increased immune response are commonly associated with an increased levels of infection. In *Aedes aegypti*, a significant augment in the level of antimicrobial peptide (AMP) transcription correlates with infectious dose of bacteria [63]. However, insect showed similar survival patterns survival as insects injected with doses of 5000 and 50,000 bacteria, even though transcription is detectable at lower concentrations [64]. In our experiment, the high concentration of IBC200614 killed insect less than the low concentration treatment in Table 1, and the muscardine cadaver rates were 95.83% and 57.24% in the populations that were killed by IMI330189 and IBC200614, respectively. In damage threshold hypothesis that was demonstrated by Moreno-García et al. in 2014, the death of insect was induced by higher fitness cost as the infection below the threshold, while the death of insect was induced by the subsequent infection of fungi as the infection above the threshold [65]. So, we speculated that the infection of low concentration of IBC200614 was below the damage threshold, and the death in insect treated by low concentration of IBC200614 was induced by higher fitness cost.

The cellular and humoral defenses are important defense mechanism in insects against infection of pathogenic fungi in the host hemolymph, and are currently being emphasized [40,66,67], because the capacity of pathogenic fungi to overcome the host hemolymph’s defenses is related to itself virulence to host [68]. IMI330189 and IBC200614 are two strains of *Metarhizium*, but they showed a significant virulence difference to locust. Possible reasons include differences in the ability to penetrate the cuticle or to overcome hemolymph immunity and propagate *in vivo*. The present study found that IMI330189 and IBC200614 could penetrate the cuticle of locust and invade hemolymph. At the beginning of infection, the yeast-like cells appeared in the two treatment groups’ hemolymph, and greatly increases of their cell were found at 72 h after inoculation, while these phenomena did not appear in the control group. These results indicated that the ability of IMI330189 and IBC200614 to penetrate the host cuticle were similarly, and suggested that the difference in the virulence of IMI330189 and IBC200614 should be caused by the difference in their ability to overcome hemocytes and humoral immunity and propagate *in vivo*.

In overcoming host hemocytes, we found that the yeast-like cells of IMI330189 converted to hemolymph-derived hyphal bodies in locust’s hemolymph at 96 h after locust was infection by IMI330189, then circulated freely in the host hemolymph or were phagocytized by host granulocytes. These hemolymph-derived hyphal bodies could grow to a size of 3–20 μm and self-reproduction in the hemolymph and granulocytes, and could gather and initiate damage locust’s hemocytes, then killed locust when their count reached peak value at 144h after locust was infected by IMI330189. At this point, IMI330189 had completely overcome the locust’s hemocytes. However, hemolymph-derived hyphal bodies of IBC200614 were not found in locust’s hemolymph at 96 h after locust was infected by IBC200614, and IBC200614 did not kill locust at 144h after locust were infected by IBC200614. This indicated that the yeast-like cells of IBC200614 strain did not convert to hemolymph-derived hyphal bodies, and IBC200614 had not overcome the locust’s hemocytes. In *M*. *anisopliae*, MCL1 gene functions as an anti-adhesive protective coat against phagocytosis and encapsulation, null mutant decrease the mortality of host by increasing the phagocytosis and encapsulation of host to *M*. *anisopliae*[69]. In IMI330189 and IBC200614, there was significantly difference in MCL1 gene because our generate primer only clone MCL1 gene from IMI330189 while it do not clone MCL1 gene from IBC200614. So, we speculated that the difference in MCL1 gene induced the difference in the colonization between IMI330189 and IBC200614, the difference in the virulence of IMI330189 and IBC200614 to locust was related with the difference in their ability to overcome locust’s hemocytes and propagate *in vivo*.

In overcoming humoral immunity, we found significant difference in the enzymes activities of locust at 7 days after locust was infected by IMI330189 or IBC200614. The increased PO activity can strengthen the immune system’s capability to challenge xenobiotics and healing in insects [36,37,39], the inhibition in PO activity caused by higher concentrations of IMI330189 suggested that the higher concentrations of IMI330189 might overcome the immunity of PO, while the increase in PO activity by lower concentration of IMI330189 and all concentration of IBC200614 suggested that the locusts might be immunized by PO. The increases in the activities of SOD, POD and CAT were related to the resistance of insects to pesticides [49,50], the increase in SOD and POD activities and the inhibition in CAT activity caused by IMI330189 suggested that IMI330189 might overcome the immunity of antioxidant enzymes in locust by inhibit the transcription of CAT, while the inhibition in SOD activity and the increase in CAT activity caused by IBC200614 suggested that IBC200614 might be immunized of antioxidant enzymes in locust by increase the transcription of CAT. GSTs play pivotal role in cellular antioxidant defenses against oxidative stress[27–29], the inhibition in GSTs/CDNB activity and the increase in GSTs/DCNB caused by IMI330189 suggested that IMI330189 might overcome the immunity of GSTs, while the increase in GSTs/CDNB and GSTs/DCNB activities caused by IBC200614 suggested that IBC200614 might be immunized by GSTs. MFOs and ESTs are two important families of enzymes in insects that participate in the detoxification of various xenobiotics and insecticides [25,26,30,31], the inhibition in MFOs and ESTs activities caused by IMI330189 in a concentration-dependence manner suggested that IMI330189 might overcome the immunity of MFOs and ESTs [70–73], while the inhibition in MFOs and ESTs activities caused by IBC200614 in non-concentration-dependence manner suggested that IBC200614 might be immunized by MFOs and ESTs [70–73]. Acetylcholinesterase (AChE) is an important enzyme at cholinergic synapses in the insect central nervous system, and is the target for organophosphate (OP) and carbamate compounds [74–76], the increase in AChE activity caused by IMI330189 and the inhibition in AChE activity caused by IBC200614 suggested that the increase of AChE activity was related to the virulence of IMI330189 to locust.

To verify the above hypothesis, we assayed the above enzymes activities of locust from the 1^st^ to 7^th^ day after spray application with a single concentration of IMI330189 or IBC200614. The no obvious impact on PO activity by IMI330189 suggested that IMI330189 might overcome the immunity provided by PO, while significant increase in PO activity by IBC200614 suggested that the insects are immunized by PO. The no obvious impact on SOD and POD activities and significant increased on CAT activity by IMI330189 suggested that IMI330189 might overcome the immunity provided by antioxidant enzymes in locust by enhance the transcription of CAT, while the significant increase on SOD and POD activities and the no obvious impact on CAT activity by IBC200614 suggested that IBC200614 enhanced the transcription of SOD and POD, increasing therefore the locust’s immune response. The significant increase on GSTs/CDNB and GSTs/DCNB activities by IMI330189 suggested that IMI330189 might overcome the immunity provided by GSTs by increase the transcription of GSTs, while significant decrease on GSTs/CDNB and GSTs/DCNB activities by IBC200614 suggested that the insects are immune to IBC200614 due to the decreased of GSTs activities. The obvious decrease on ESTs activities by IMI330189 suggested that IMI330189 might overcome the immunity of ESTs by inhibit the transcription of ESTs, while the no obvious impact on ESTs activities by IBC200614 suggested that the insects are immune to IBC200614 due to the stability of ESTs activities. The significant decrease on MFOs and AChEs activities by IMI330189 suggested that IMI330189 might overcome the immunity of MFOs and AChEs by inhibit the transcription of MFOs and AChEs, while the significant increase on MFOs and AChEs activities by IBC20016 suggested that the insects are immune to IBC200614 due to the increase of MFOs and AChEs activities. The significant increase on AA activity by IMI330189 suggested that IMI330189 might overcome the immunity of locust by increase the transcription of AA, while the no obvious impact on AA by IBC200614 suggested that insects are immune to IBC200614 due to the stability of AA activities. So, IMI330189 could overcome the immune of locust by decrease the activities of PO, AChEs, ESTs and MFOs while increase the activities of CAT, AA and GSTs. However, locust could immune to IBC200614 by increase the activities of PO, MFOs, AChEs, SOD and POD while decrease the activities of GSTs and the stability of AA and ESTs activities. We speculated that the difference in above enzymes activities changes was induced by the difference in secretions between IMI330189 and IBC200614 because that the difference in above enzymes activities changes was mainly found in infection process and *M*. *anisopliae* could initiate difference variation in SOD, CAT and POD in difference insect [52,53]. In *P*. *Americana*, *M*. *anisopliae* fungal infections initiate oxidative stress in the midgut, fat body, whole body and hemolymph, and decrease the CAT activity of fat body and midgut [52]. In *S*. *litura*, the activities of SOD, CAT and POD in the larval body decreased after it was treated with crude destruxins at the doses that caused 90% mortality [53].

In previous study, the three days after spray application of the fungi has been known as the early stage of infection, and the detoxifying ESTs and GSTs may participate in the defense reactions of insect to *Metarihizium* [32]. In this study, we found that the period from the second day to the fifth day after spray of *Metarizhizium* was the most important stage of virulence, the changes in enzymes activity was significantly associated to the virulence of *Metarizhizium*. The lower virulence strain IBC200614 significantly increased the activities of SOD, PO and MFOs on the 2^nd^ day after spray application, and significantly increased the activities of CAT and CHI on the 3^rd^ day after spray application. The higher virulence strain IMI330189 slightly increased the activity of PO on the 2^nd^ day after spray application, and slightly increased the activities of SOD and POD on the 3^rd^ day after spray application. On the 4^th^ day after spray, IBC200614 significantly increased the activities of PO, MFOs, SOD and CAT, while IMI330189 increased the activities of PO and POD. On the 5^th^ day after spray, IBC200614 increased the activities of PO, MFOs, SOD and CAT, and IMI330189 increased the activity of POD. So, the period from the second day to fifth day after spray of *Metarizhizium* was the most important stage of virulence.

The above results showed that the higher virulence strain IMI330189 could overcome the hemocytes and humoral immunity by decrease the activities of PO, AChEs, ESTs and MFOs and increase the activities of CAT, AA and GSTs, and the period from the second day to the fifth day after spray of *M*. *anisopliae* was the most important stage of virulence. These results provide insights that can be used to strengthen the virulence of *M*. *anisopliae* and aid in selecting the optimum insecticides to rotate or mix with *M*. *anisopliae*.

## Supporting Information

S1 Table Fold changes in the activities of biochemical enzymes of *L*. *migratoria* on the 7th day after it was treated by different concentrations of *M*. *anisopliae* strains IMI330189 and IBC200614. Fold changes of enzymes activities were showed as means±SD. Means (±SD) followed by different lowercase letters within row of one enzyme are significantly different by Tukey’s HSD (*P* < 0.05).
(DOCX)

S2 Table Correlation analysis of the logarithm of *M*. *anisopliae* strain IMI330189 concentrations and enzyme activities during infection of *L*. *migratoria*. R is an abbreviation of the correlation coefficient.
(DOCX)

S3 Table Correlation analysis of the logarithm of *M*. *anisopliae* strain IBC200614 concentrations and enzyme activities during infection of *L*. *migratoria*. R is an abbreviation of the correlation coefficient.
(DOCX)

S4 Table Fold changes in the activities of biochemical enzymes of *L*. *migratoria* during the infection process of *M*. *anisopliae* strains IMI330189 and IBC200614 at similar concentrations. IMI is an abbreviation of IMI330189, IBC is an abbreviation of IBC200614. Fold changes of enzymes activities were showed as means±SD. Means (±SD) followed by different lowercase letters within columns of one enzyme are significantly different by Tukey’s HSD (*P* < 0.05).
(DOCX)
