# Correction: Different Effects of *Metarhizium anisopliae* Strains IMI330189 and IBC200614 on Enzymes Activities and Hemocytes of *Locusta migratoria* L.

**DOI:** 10.1371/journal.pone.0175219

**Published:** 2017-03-30

**Authors:** Guangchun Cao, Miao Jia, Xia Zhao, Lei Wang, Xiongbing Tu, Guangjun Wang, Xiangqun Nong, Zehua Zhang

The funding statement and acknowledgments section incorrectly reference the grant number from the National Natural Science Foundation of China. The correct grant should read: National Natural Science Foundation of China, 31471824.
