# Supplementary material for: Different Effects of Metarhizium anisopliae Strains IMI330189 and IBC200614 on Enzymes Activities and Hemocytes of Locusta migratoria L
Source: PLoS One. 2016 May 26;11(5):e0155257. doi: 10.1371/journal.pone.0155257 (PMC4881918; doi:10.1371/journal.pone.0155257)
Supplement: S1 Table — Fold changes of enzymes activities were showed as means±SD. Means (±SD) followed by different lowercase letters within row of one enzyme are significantly different by Tukey’s HSD (P < 0.05). (DOCX) [file pone.0155257.s001.docx]

**S1Table.** Fold changes in the activities of biochemical enzymes of *L. migratoria* on the 7^th^ day after it was treated by different concentrations of *M. anisopliae* strains IMI330189 and IBC200614.

| Enzyme | Metarhizium | Fold changes of enzymes activities | | | | | |
| --- | --- | --- | --- | --- | --- | --- | --- |
|  |  | ck | 1×10^3^ | 1×10^4^ | 1×10^5^ | 1×10^6^ | 1×10^7^ |
| ESTs | IMI330189 | 1.00±0.006 a | 0.77±0.001 b | 0.66±0.002 e | 0.68±0.003 d | 0.65±0.004 f | 0.52±0.001 h |
|  | IBC200614 | 1.00±0.006 a | 0.47±0.002 i | 0.55±0.004 g | 0.70±0.006 c | 0.77±0.003 b | 0.77±0.003 b |
| AChEs | IMI330189 | 1.00±0.020 cd | 1.01±0.006 cd | 1.06±0.015 b | 1.02±0.020 bc | 1.16±0.005 a | 1.15±0.009 a |
|  | IBC200614 | 1.00±0.020 cd | 0.90±0.009 f | 1.06±0.014 b | 0.92±0.008 f | 0.94±0.023 ef | 0.97±0.027 de |
| MFOs | IMI330189 | 1.00±0.129 a | 0.87±0.115 ab | 0.86±0.042 ab | 0.59±0.099 c | 0.38±0.066 d | 0.69±0.016 bc |
|  | IBC200614 | 1.00±0.129 a | 0.74±0.015 bc | 0.79±0.014 b | 0.23±0.032 d | 0.37±0.018 d | 0.37±0.018 d |
| PO | IMI330189 | 1.00±0.008 d | 0.98±0.001 d | 2.66±0.010 b | 0.41±0.006 h | 0.47±0.017 g | 0.39±0.005 h |
|  | IBC200614 | 1.00±0.008 d | 1.69±0.013 c | 0.89±0.015 e | 6.95±0.048 a | 0.71±0.002 f | 0.65±0.022 f |
| GSTs/CDNB | IMI330189 | 1.00±0.009 f | 0.93±0.002 h | 0.84±0.003 i | 0.84±0.004 i | 0.96±0.002 g | 0.96±0.004 g |
|  | IBC200614 | 1.00±0.009 f | 1.53±0.004 a | 1.17±0.008 e | 1.39±0.005 b | 1.31±0.007 c | 1.26±0.001 d |
| GSTs/DCNB | IMI330189 | 1.00±0.023 h | 0.86±0.029 i | 1.22±0.044 g | 1.45±0.043 e | 1.53±0.006 de | 1.54±0.040 d |
|  | IBC200614 | 1.00±0.023 h | 1.66±0.069 c | 1.06±0.025 h | 2.18±0.023 a | 1.33±0.018 f | 1.84±0.004 b |
| SOD | IMI330189 | 1.00±0.005 de | 1.07±0.005 a | 1.08±0.009 a | 1.04±0.018 bc | 1.04±0.013 b | 1.01±0.014 cd |
|  | IBC200614 | 1.00±0.005 de | 0.92±0.012 hi | 0.97±0.003 fg | 0.94±0.006 gh | 0.98±0.002 ef | 0.91±0.006 i |
| CAT | IMI330189 | 1.00±0.004 d | 1.05±0.001 bc | 0.65±0.004 g | 1.12±0.005 a | 0.90±0.020 e | 1.03±0.001 c |
|  | IBC200614 | 1.00±0.004 d | 1.03±0.001 c | 1.06±0.001 b | 0.88±0.002 f | 1.00±0.002 d | 1.11±0.002 a |
| POD | IMI330189 | 1.00±0.005 cd | 1.09±0.005 a | 1.06±0.026 ab | 1.04±0.035 bc | 1.07±0.010 ab | 1.00±0.013 cde |
|  | IBC200614 | 1.00±0.005 cd | 1.05±0.008 ab | 0.96±0.005 e | 0.96±0.006 de | 1.04±0.007 b | 1.00±0.002 cd |
| CHI | IMI330189 | 1.00±0.033 abc | 0.93±0.058abc | 1.00±0.180 abc | 1.04±0.102abc | 1.18±0.039 a | 0.92±0.153 abc |
|  | IBC200614 | 1.00±0.033 abc | 0.76±0.060 c | 1.09±0.013 ab | 1.12±0.022 ab | 0.84±0.163 bc | 0.86±0.086 bc |
| AA | IMI330189 | 1.00±0.011 d | 2.37±0.038 a | 1.04±0.011 d | 1.17±0.102 bc | 0.73±0.004 e | 0.99±0.010 d |
|  | IBC200614 | 1.00±0.011 d | 0.73±0.007 e | 1.08±0.068 cd | 1.24±0.013 b | 0.75±0.007 e | 0.82±0.038 e |

Fold changes of enzymes activities were showed as means±SD. Means (±SD) followed by different lowercase letters within row of one enzyme are significantly different by Tukey’s HSD (*P* < 0.05).
