# Supplementary material for: Different Effects of Metarhizium anisopliae Strains IMI330189 and IBC200614 on Enzymes Activities and Hemocytes of Locusta migratoria L
Source: PLoS One. 2016 May 26;11(5):e0155257. doi: 10.1371/journal.pone.0155257 (PMC4881918; doi:10.1371/journal.pone.0155257)
Supplement: S2 Table — R is an abbreviation of the correlation coefficient. (DOCX) [file pone.0155257.s002.docx]

**S2Table.** Correlation analysis of the logarithm of *M. anisopliae* strain IMI330189 concentrations and enzyme activities during infection of *L. migratoria.*

| Enzyme | The equation | R | *P*-value |
| --- | --- | --- | --- |
| GSTs/DCNB | *y*=7.40*x*+21.70 | 0.92 | 0.03 |
| SOD | *y*=-0.64*x*+42.31 | 0.9 | 0.03 |
| ESTs | *y*=-1039.40*x*+18333.00 | 0.90 | 0.04 |
| AChEs | *y*=4.54*x*+102.70 | 0.85 | 0.07 |
| POD | *y*=-1.53*x*+92.63 | 0.83 | 0.08 |
| AA | *y*=-0.28*x*+2.59 | 0.76 | 0.14 |
| MFOs | *y*=-1.10*x*+13.80 | 0.69 | 0.20 |
| PO | *y*=-10.72*x*+84.72 | 0.55 | 0.34 |
| GSTs/CDNB | *y*=141.88*x*+6215.07 | 0.50 | 0.41 |
| CHI | *y*=0.05*x*+3.17 | 0.22 | 0.72 |
| CAT | *y*=5.63*x*+218.14 | 0.19 | 0.76 |

R is an abbreviation of the correlation coefficient
