# Supplementary material for: Different Effects of Metarhizium anisopliae Strains IMI330189 and IBC200614 on Enzymes Activities and Hemocytes of Locusta migratoria L
Source: PLoS One. 2016 May 26;11(5):e0155257. doi: 10.1371/journal.pone.0155257 (PMC4881918; doi:10.1371/journal.pone.0155257)
Supplement: S3 Table — R is an abbreviation of the correlation coefficient. (DOCX) [file pone.0155257.s003.docx]

**S3Table.** Correlation analysis of the logarithm of *M. anisopliae* strain IBC200164 concentrations and enzyme activities during infection of *L. migratoria.*

| Enzyme | The equation | | R | *P*-value |
| --- | --- | --- | --- | --- |
| ESTs | | *y*=1618.30*x*+4958.27 | 0.95 | 0.05 |
| MFOs | | *y*=-1.44*x*+13.38 | 0.74 | 0.15 |
| GSTs/CDNB | | *y*=-210.67*x*+11063 | 0.25 | 0.68 |
| GSTs/DCNB | | *y*=2.72*x*+58.26 | 0.22 | 0.72 |
| CAT | | *y*=2.39*x*+251.68 | 0.17 | 0.79 |
| PO | | *y*=-7.18*x*+105.02 | 0.13 | 0.83 |
| AA | | *y*=-0.01*x*+0.92 | 0.10 | 0.88 |
| AChEs | | *y*=0.32*x*+109.34 | 0.07 | 0.91 |
| POD | | *y*=-0.12*x*+81.45 | 0.06 | 0.93 |
| CHI | | *y*=-0.02*x*+3.26 | 0.06 | 0.92 |
| SOD | | *y*=-0.01*x*+35.19 | 0.01 | 0.98 |

R is an abbreviation of the correlation coefficient
