# Supplementary material for: Different Effects of Metarhizium anisopliae Strains IMI330189 and IBC200614 on Enzymes Activities and Hemocytes of Locusta migratoria L
Source: PLoS One. 2016 May 26;11(5):e0155257. doi: 10.1371/journal.pone.0155257 (PMC4881918; doi:10.1371/journal.pone.0155257)
Supplement: S4 Table — IMI is an abbreviation of IMI330189, IBC is an abbreviation of IBC200614. Fold changes of enzymes activities were showed as means±SD. Means (±SD) followed by different lowercase letters within columns of one enzyme are significantly different by Tukey’s HSD (P < 0.05). (DOCX) [file pone.0155257.s004.docx]

**S4Table.** Fold changes in the activities of biochemical enzymes of *L. migratoria* during the infection process of *M. anisopliae* strains IMI330189 and IBC200614 at similar concentrations.

| enzymes |  | Fold changes of enzymes activities | | | | | | | |
| --- | --- | --- | --- | --- | --- | --- | --- | --- | --- |
|  |  | 0 | 1 | 2 | 3 | 4 | 5 | 6 | 7 |
| ESTs | CK | 1.00±0.001a | 1.00±0.001b | 1.00±0.001a | 1.00±0.004a | 1.00±0.001b | 1.00±0.002b | 1.00±0.001b | 1.00±0.005a |
|  | IMI | 1.00±0.006a | 0.84±0.007c | 0.57±0.009c | 0.94±0.008c | 0.78±0.001c | 1.06±0.003a | 0.79±0.003c | 0.69±0.003c |
|  | IBC | 1.00±0.002a | 1.10±0.018a | 0.71±0.007b | 0.97±0.003b | 1.09±0.005a | 0.93±0.001c | 1.05±0.004a | 0.97±0.002b |
| GSTs/CDNB | CK | 1.00±0.013a | 1.00±0.014b | 1.00±0.016a | 1.00±0.016b | 1.00±0.018c | 1.00±0.009b | 1.00±0.025b | 1.00±0.015a |
|  | IMI | 1.00±0.013a | 0.95±0.004c | 0.93±0.014b | 1.56±0.016a | 1.44±0.012a | 1.06±0.009a | 1.59±0.016a | 0.93±0.011b |
|  | IBC | 1.00±0.013a | 1.09±0.001a | 0.71±0.021c | 0.70±0.008c | 1.29±0.016b | 0.88±0.011c | 0.58±0.006c | 0.80±0.007c |
| GSTs/DCNB | CK | 1.00±0.016a | 1.00±0.016a | 1.00±0.054a | 1.00±0.049a | 1.00±0.056b | 1.00±0.011b | 1.00±0.085b | 1.00±0.027a |
|  | IMI | 1.00±0.016a | 1.00±0.030a | 0.94±0.037a | 0.92±0.030b | 1.26±0.049a | 1.38±0.048a | 1.32±0.049a | 0.92±0.029b |
|  | IBC | 1.00±0.016a | 1.01±0.054a | 0.77±0.014b | 0.53±0.038c | 0.97±0.023b | 0.77±0.017c | 0.82±0.015c | 1.04±0.045a |
| MFOs | CK | 1.00±0.016a | 1.00±0.019a | 1.00±0.057b | 1.00±0.039a | 1.00±0.053b | 1.00±0.009b | 1.00±0.044a | 1.00±0.117a |
|  | IMI | 1.00±0.016a | 0.99±0.061a | 0.99±0.076b | 0.79±0.087b | 0.85±0.027c | 0.62±0.042c | 0.61±0.025b | 0.97±0.058a |
|  | IBC | 1.00±0.016a | 0.97±0.104a | 1.48±0.133a | 0.67±0.019c | 2.61±0.057a | 2.53±0.024a | 1.02±0.003a | 1.06±0.005a |
| AChEs | CK | 1.00±0.015a | 1.00±0.016b | 1.00±0.008b | 1.00±0.010a | 1.00±0.024b | 1.00±0.032a | 1.00±0.028a | 1.00±0.025a |
|  | IMI | 1.00±0.015a | 1.01±0.017b | 1.28±0.018a | 0.55±0.031c | 1.09±0.016a | 0.90±0.043b | 0.78±0.043b | 0.87±0.041b |
|  | IBC | 1.00±0.015a | 1.22±0.022a | 0.88±0.026c | 0.67±0.039b | 0.89±0.027c | 0.63±0.062c | 0.61±0.025c | 0.78±0.035c |
| PO | CK | 1.00±0.026a | 1.00±0.027a | 1.00±0.056c | 1.00±0.058c | 1.00±0.030c | 1.00±0.020b | 1.00±0.011b | 1.00±0.011b |
|  | IMI | 1.00±0.026a | 0.95±0.078a | 1.83±0.186b | 1.78±0.036b | 1.47±0.022b | 0.84±0.044c | 0.89±0.005c | 0.54±0.002c |
|  | IBC | 1.00±0.026a | 0.99±0.036a | 3.48±0.006a | 6.42±0.055a | 5.09±0.067a | 3.12±0.013a | 4.16±0.005a | 1.77±0.008a |
| SOD | CK | 1.00±0.0003a | 1.00±0.001c | 1.00±0.0004b | 1.00±0.0002c | 1.00±0.0004a | 1.00±0.001b | 1.00±0.001b | 1.00±0.001b |
|  | IMI | 1.00±0.0003a | 1.10±0.0004b | 0.72±0.001c | 1.86±0.005b | 0.95±0.0004b | 0.64±0.001c | 0.58±0.001c | 1.21±0.001a |
|  | IBC | 1.00±0.0003a | 1.17±0.017a | 2.73±0.0004a | 2.09±0.0004a | 1.03±0.0004a | 1.66±0.0003a | 1.21±0.001a | 0.84±0.0004c |
| CAT | CK | 1.00±0.0002a | 1.00±0.0002a | 1.00±0.0002a | 1.00±0.0002b | 1.00±0.0002c | 1.00±0.0002c | 1.00±0.0002b | 1.00±0.0001b |
|  | IMI | 1.00±0.0002a | 0.93±0.0002c | 0.86±0.0001c | 1.12±0.018a | 1.65±0.098a | 1.28±0.0004a | 1.40±0.0002a | 1.08±0.0003a |
|  | IBC | 1.00±0.0002a | 0.96±0.0003b | 0.97±0.0001b | 0.94±0.0001c | 1.03±0.0001b | 1.07±0.0002b | 0.75±0.0002c | 0.99±0.0004c |
| POD | CK | 1.00±0.0001a | 1.00±0.0001a | 1.00±0.0005a | 1.00±0.0003b | 1.00±0.0005b | 1.00±0.0005b | 1.00±0.0002a | 1.00±0.0002c |
|  | IMI | 1.00±0.0001a | 0.98±0.0004b | 0.95±0.0004b | 0.91±0.002c | 0.66±0.0003c | 0.65±0.0002c | 0.89±0.001b | 1.48±0.0002b |
|  | IBC | 1.00±0.0001a | 1.00±0.0003a | 0.80±0.0003c | 1.53±0.0004a | 1.45±0.001a | 1.17±0.0001a | 0.83±0.0001c | 1.85±0.0002a |
| AA | CK | 1.00±0.011a | 1.00±0.013a | 1.00±0.003b | 1.00±0.006b | 1.00±0.007a | 1.00±0.016b | 1.00±0.010b | 1.00±0.009b |
|  | IMI | 1.00±0.011a | 0.99±0.004a | 1.17±0.006a | 0.97±0.004c | 1.00±0.006a | 1.15±0.008a | 1.10±0.017a | 1.14±0.012a |
|  | IBC | 1.00±0.011a | 1.00±0.017a | 0.77±0.013c | 1.03±0.017a | 0.99±0.016a | 0.95±0.011c | 0.93±0.012c | 0.86±0.018c |
| CHI | CK | 1.00±0.017a | 1.00±0.018a | 1.00±0.027a | 1.00±0.064c | 1.00±0.014b | 1.00±0.011b | 1.00±0.021b | 1.00±0.008b |
|  | IMI | 1.00±0.017a | 1.00±0.030a | 0.89±0.017b | 1.09±0.020b | 0.95±0.013c | 1.01±0.014b | 0.89±0.019c | 1.00±0.014b |
|  | IBC | 1.00±0.017a | 1.00±0.071a | 0.85±0.026b | 1.30±0.036a | 1.11±0.033a | 1.27±0.176a | 1.11±0.004a | 1.17±0.055a |

IMI is an abbreviation of IMI330189, IBC is an abbreviation of IBC200614

Fold changes of enzymes activities were showed as means±SD.

Means (±SD) followed by different lowercase letters within columns of one enzyme are significantly different by Tukey’s HSD (*P* < 0.05).
